# Non-Adherence to Anti-Tuberculosis Treatment and Determinant Factors among Patients with Tuberculosis in Northwest Ethiopia

**DOI:** 10.1371/journal.pone.0078791

**Published:** 2013-11-11

**Authors:** Akilew Awoke Adane, Kefyalew Addis Alene, Digsu Negese Koye, Berihun Megabiaw Zeleke

**Affiliations:** Institute of Public Health, College of Medicine and Health Sciences, University of Gondar, Gondar, Ethiopia; Institute of Infectious Diseases and Molecular Medicine, South Africa

## Abstract

**Background:**

Non-adherence to anti tuberculosis treatment is one of the crucial challenges in improving tuberculosis cure-rates and reducing further healthcare costs. The poor adherence to anti-tuberculosis treatment among patients with tuberculosis is a major problem in Ethiopia. Hence, this study assessed level of non-adherence to anti-tuberculosis therapy and associated factors among patients with tuberculosis in northwest Ethiopia.

**Methods:**

An institution based cross-sectional survey was conducted among tuberculosis patients who were following anti-tuberculosis treatment in North Gondar zone from February 20 – March 30, 2013. Data were collected by trained data collectors using a structured and pre-tested questionnaire. Data were entered to EPI INFO version 3.5.3 and analyzed using statistical package for social sciences (SPSS) version 20. Multiple logistic regressions were fitted to identify associations and to control potential confounding variables. Odds ratio (OR) with 95% confidence interval was calculated and p-values<0.05 were considered statistically significant.

**Results:**

A total of 280 tuberculosis patients were interviewed; 55.7% were males and nearly three quarters (72.5%) were urban dwellers. The overall non-adherence for the last one month and the last four days before the survey were 10% and 13.6% respectively. Non-adherence was high if the patients had forgetfulness (AOR 7.04, 95% CI 1.40–35.13**),** is on the continuation phase of chemotherapy (AOR: 6.95, 95% CI 1.81–26.73), had symptoms of tuberculosis during the interview (AOR: 4.29, 95% CI 1.53–12.03), and had co-infection with HIV (AOR: 4.06, 95% CI 1.70–9.70).

**Conclusions:**

Non-adherence to anti-tuberculosis treatment was high. Forgetfulness, being in the continuation phases of chemotherapy, having symptoms of tuberculosis during the interview, and co-infected with HIV were significantly associated with non-adherence to anti-tuberculosis therapy. Special attention on adherence counseling should be given to symptomatic patients, TB/HIV co-infected patients, and those in the continuation phase of the tuberculosis therapy.

## Introduction

Tuberculosis (TB), one of the oldest diseases known to affect humans, is a major cause of death worldwide. It usually affects the lungs, although other organs are involved in up to one-third of the cases. If left untreated, 50–65% of TB cases will die within 5 years [1]. In 2010, there were 8.8 million new cases of TB and 1.45 million deaths from TB worldwide. The human immunodeficiency virus (HIV) pandemic presents a significant challenge to global TB control. TB is the second leading cause of death from an infectious disease worldwide among all people and the leading preventable cause of death among people living with HIV. About 13% of TB cases occur among people living with HIV [2], [3].

According to recent estimates, Ethiopia stands 7^th^ in the list of high TB burden countries. In Ethiopia, TB is the leading cause of morbidity, the third cause of hospital admission, and the second cause of death. The estimated TB incidence in Ethiopia was 261/ 100,000 inhabitants in 2011. The lifetime risk of developing TB in Ethiopia is estimated to be 50–60% for HIV-infected people, only 10% for HIV-negative counterparts [3], [4].

Poor adherence to treatment of chronic diseases including TB is a worldwide problem of striking magnitude [5]. However, patients with TB are expected to have adherence levels greater than 90% in order to facilitate cure [6], [7]. The failure for cure increases the risk of development of drug resistant strains and further spread of TB in the community, which in turn increases morbidity and mortality. Adherence to TB treatment is crucial to achieve cure and avoid emergence of drug resistance [7]. In sub Saharan Africa, there is high rate of losses to follow up of TB patients that ranged from 11.3% to 29.6% [8]. Ethiopia is one of the seven countries that reported lower rates of treatment success (84%) [3]. Patients who take their TB treatment in an irregular and unreliable way are at greatly increased risk of treatment failure, relapse and the development of drug-resistant TB strains. Furthermore, the emergence and spread of multi drug resistant (MDR) and extensive drug resistant (XDR) TB further reinforces the absolute necessity of helping TB patients to not miss any doses of anti TB [9], [10]. However, it is very difficult to have regular supervision and support of TB patients in developing countries like Ethiopia. Hence, this study assessed the level of non-adherence to anti-TB therapy and associated factors among TB patients in northwest Ethiopia.

## Methods

### Study Setting

The study was conducted in the North Gondar zone, northwest Ethiopia. The zone has 22 districts with a total population of 2,921,470. All health facilities providing anti-TB services in three major towns of north Gondar (Gondar, Metema and Debark) were included. Newly diagnosed TB patients receive six months of standard TB chemotherapy in two phases- an intensive phase for two months and a continuation phase of four months. A fixed dose combination of RHZE (Rifampicin, Isoniazid, Pirazinamide and Ethambutol) is given in the intensive phase of anti-TB therapy while only RH during the continuation phase. Patients collect anti-TB drugs daily during the intensive phase except weekends while weekly or monthly during the continuation phase. Provider initiated voluntary counseling and testing for HIV infection was also offered currently to all TB patients.

### Study Design and Participants

An institution based cross-sectional survey was conducted from February 20 – March 30, 2013. Eligible patients were new TB patients aged at least 15 years regardless of the site or the smear status of their TB. To be eligible for this study, patients must have also taken anti-TB at least for a month.

### Sample Size and Sampling Procedures

The sample size was calculated using a single population proportion formula; considering 21% proportion of non- adherence level according to previous similar study from southwest Ethiopia (16), a 5% margin of error, and 10% possible non-response rate. The sample size was estimated to be 281. All adult TB patients (≥15 years old) on anti-TB at tuberculosis follow up clinics in the three (Gondar, Metema and Dabark) towns within the study period were interviewed.

### Data Collection and Assessment of Non - adherence

Data were collected using an interviewer-administered questionnaire. The questionnaire was composed of socio-demographic characteristics, patient related; therapy or drug related information, social and health system /health provider related variables and pre-tested before the actual data collection. Data were collected after verbal informed consent was obtained. Six nurses who were trained for this purpose collected the data. Data collectors were also not part of the health facilities where TB patients interviewed.

Recent non- adherence (last 4 days) and non-adherence during the last one month before the survey were assessed. Patients were asked to report the total number of anti-TB pills they missed 4 days before the survey. These numbers of pills were compared to the number of pills prescribed to the patient. Adherence to TB medication in the last 4 days was classified as non-adherent (missed at least 1 (25%) of the pills prescribed over 4 days) and completely adherent (no missed pill in the last 4 days before the survey). Similarly, the last one month non-adherence was calculated as total reported missed pills over the total prescribed pills within that month and classified as adherent (no more than 10% of pills missed) and otherwise non- adherent. Patients were also asked to report why they missed anti-TB drugs. A patient who scores three or more than three from a six item questions asked to measure TB knowledge were considered to have good knowledge about TB. These questions were obtained from information routinely provided to patients as part of the national TB program.

### Data Analysis

Data were entered to EPI INFO version 3.5.3 and analysis was conducted using statistical package for social sciences (SPSS) version 20. Descriptive statistics like frequencies and cross tabulation were made for most selected variables. Multiple logistic regression analysis was used to assess the association between the dependent variable and each independent variable. Variables with p-values ≤0.2 in bivariate analysis were fitted in the final multiple logistic regression model to assess the strength of association and control confounding effects. Both Crude Odds Ratio (COR) and Adjusted Odds Ratio (AOR) with 95% confidence interval (CI) were used to show an association between selected variables. Variables having p-value ≤0.05 in the final model were taken as significant determinants.

### Ethical Considerations

The ethical clearance was obtained from the Institutional Review Board of the University of Gondar. The Institutional Review Board approved the oral informed consent since it was anticipated that many of the study subjects could not read and write. In addition to this, the Institutional Review Board approved all ethical procedures, based on the awareness about the type of study (that it was harmless to study subjects) and the education level of study subjects. An official permission letter was obtained from each health facility administration office. During data collection, oral informed consent was obtained from all participants after they were introduced to the purpose of the study and informed about their rights to interrupt the interview at any time. To ensure confidentiality, names were avoided in the questionnaire and reporting the results of the study. In addition, the collected information was locked with a key (hard copies) and by passwords (soft copies). Informed oral consent was also obtained from parents or guardians for subjects under 18 years old.

## Results

### Socio-demographic Characteristics

A total of 280 TB patients (with a response rate of 99.6%) were interviewed. Nearly three quarters (72.5%) were urban dwellers and had a mean age of 32.9 (±14.8 SD) years. About half of them (50.4%) did not attend any formal education and nearly one fifth of them were farmers. Amhara (98.2%) and Orthodox Christianity (89.6%) were the most common ethnicity and religion respectively (Table 1).

**Table 1:** Socio-demographic characteristics of respondents’, North Gondar zone, Northwest Ethiopia, May 2013 (n = 280).

Characteristics		Frequency	Percent
Residence	Urban	203	72.5
	Rural	77	27.5
Sex	Male	156	55.7
	Female	124	44.3
Age (years)	≤32	171	61.1
	>32	109	38.9
Marital status	Single	121	43.2
	Married	118	42.1
	Divorced	31	11.1
	Widowed	10	3.6
Education level	No formal education	141	50.4
	Primary level	85	30.4
	Secondary level &above	54	19.3
Occupation	Farmer	58	20.7
	Merchant	48	17.1
	Daily laborer	43	15.4
	Housewife	48	17.1
	Students	39	13.9
	Government employee	17	6.1
	Jobless	19	6.8
	Others	8	2.9
Religion	Orthodox	251	89.6
	Muslim	29	10.4
Ethnicity	Amhara	275	98.2
	Tigrie	5	1.8

### Non-adherence to Anti-TB Treatment and Reasons of Pill Missing

**Figure 1:**
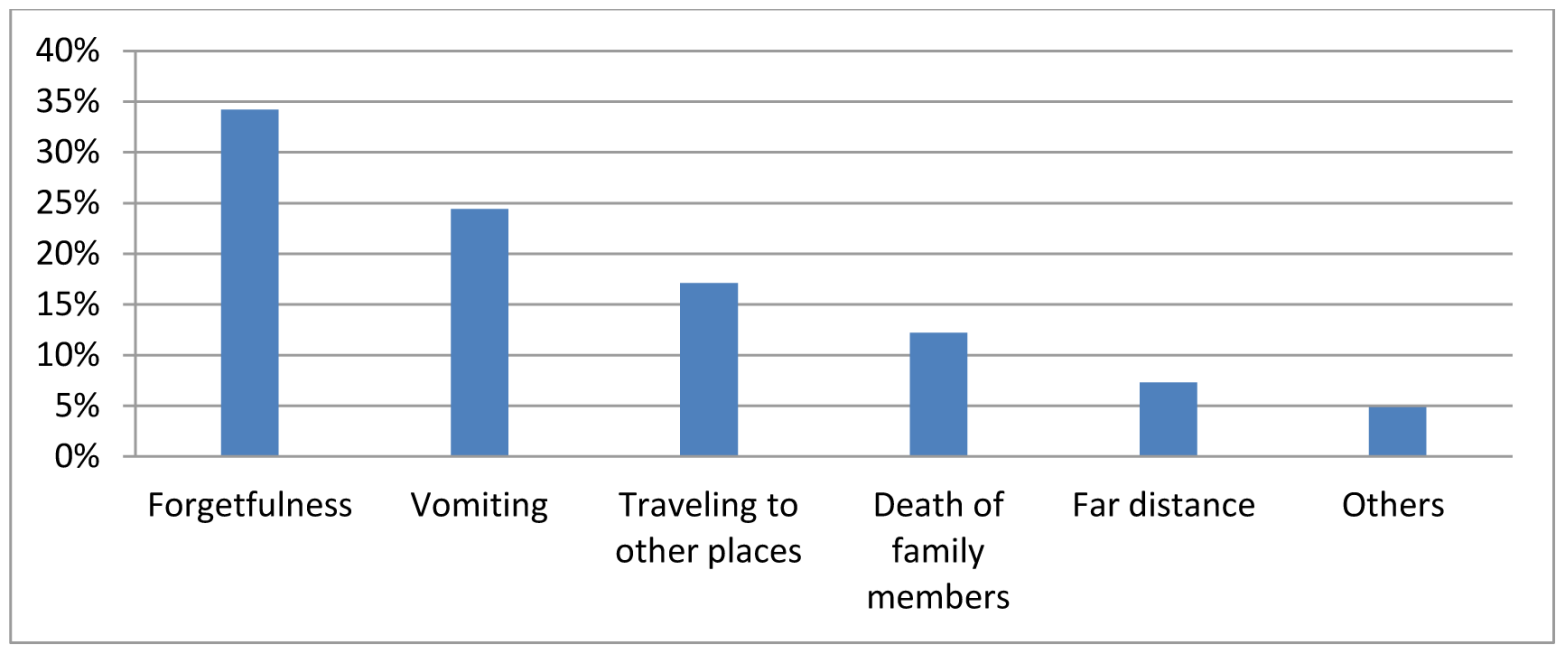
Reasons of anti-TB pills missing, North Gondar zone, Northwest Ethiopia, May 2013.

The overall calculated non-adherence over one month and the last four days before the survey were 10% and 13.6% respectively. Forgetfulness (34%), vomiting (24%) and traveling to other places (17%) were the most frequent reasons for missing the anti-TB pills (Figure 1).

### Patient Related Characteristics

About 6% of TB patients reported that they had a problem of forgetfulness. Almost all (98.9%) participants had good knowledge about TB but 3.6% of participants reported they had stopped taking their medication without telling their health care provider because they felt worse when they took it. Similarly, 3.6% of participants reported that they stopped taking their anti-TB medication when they felt better. About 6% of participants were substance users.

### Healthcare System and Other Related Characteristics

Ninety five percent of TB patients walked to and from the health facilities and 26.8% of participants reported that it took them more than half an hour to reach the nearby health facility providing them the anti-TB drugs. Nearly half of participants (54.3%) reported that the health workers were very friendly to them.

Most of TB patients (97.1%) in the current study disclosed their illness to their relatives and most (75%) of those who had not disclosed were due to fear of stigma. The rest had no relatives with whom to share their problem. About one in eight (12.9%) participants visited quacks (i.e. Traditional healers) while they were taking their chemotherapy (Table 2).

**Table 2:** Healthcare system and other related characteristics of respondents’, North Gondar zone, Northwest Ethiopia, May 2013.

Characteristics		Frequency	Percent
Time to reach to the health facility	≤30 minutes	205	73.2
	>30 minutes	75	26.8
Transportation	On foot	266	95
	Public transport	14	5.0
Waiting time in the health facilities	≤30 minutes	269	96.1
	>30 minutes	11	3.9
Availability of drugs	Always available	278	99.3
	Not always available	2	0.7
Counseling	Yes	204	72.9
	No	76	27.1
Relationship with health workers	Very friendly	152	54.3
	Friendly	126	45.0
	Unfriendly	2	0.7
TB status disclosure to the family	Yes	272	97.1
	No	8	2.9
Treatment supporter	Health facility worker	166	59.3
	Health extension worker	4	1.4
	A family member	182	65
	Religious leaders	10	3.6
	No treatment supporter	22	7.9
Visited quacks	Yes	36	12.9
	No	244	87.1

Quacks are ‘Traditional healers’.

### TB Disease and Treatment Related Characteristics

A significant proportion of TB patients (38.9%) reported some kind of anti-TB medication adverse effects. Three quarters (75.2%) and 37% of participants complained of minor adverse effects such as urine discolorations and headache or dizziness, respectively. Most patients (87.5%) were naive for anti-TB medication. Almost all (97.5%) of the TB patients were screened for HIV and nearly one quarter (23.4%) of them were positive (Table 3).

**Table 3:** Anti-TB therapy and diseases related characteristics of TB patients, North Gondar zone, Northwest Ethiopia, May 2013.

Characteristics		Frequency	Percent
Experience of side effects	Yes	109	38.9
	No	171	61.1
Types of side effects (n = 109)	Skin rash	17	15.6
	Headache & dizziness	42	36.7
	Yellow eyes	13	11.9
	Vomiting	32	29.4
	Urine discoloration	82	75.2
Symptoms of TB during the interview	Yes	36	12.9
	No	244	87.1
Treatment category	New	245	87.5
	Re-treatment	35	12.5
Types of TB	PTB-SM+	116	41.4
	PTB-SM−	97	34.7
	EPTB	67	23.9
HIV screening status	Screened	273	97.5
	Not screened	7	2.5
HIV status (n = 273)	Positive	64	23.4
	Negative	209	76.6
ART status (n = 64)	Started	44	68.8
	Not started	20	31.2
OIs other than TB (n = 64)	Yes	17	26.6
	No	47	73.4
Drugs other than anti-TB & HAART (n)	None	221	93.6
	CPT	15	6.4
Phases of chemotherapy	Intensive phase	86	30.7
	Continuation phase	194	69.3

CPT = cotrimoxazole preventive therapy, EPTB = Extra pulmonary TB, PTB-SM+ = Smear positive pulmonary TB, PTB-SM− = Smear negative pulmonary TB, OI = Opportunistic Infections.

### Factors Associated with Non-adherence to Anti-TB Treatment

In the bivariate analysis, forgetfulness, phases of chemotherapy, symptoms of TB during the interview, HIV co-infection, experience of drug side-effects, visiting quacks, and taking additional drugs other than anti-TB were significantly associated with anti-TB treatment non-adherence.

However, in the adjusted analysis, forgetfulness, phases of chemotherapy, symptoms of TB during the interview and HIV co-infection were remained significantly and independently associated with anti-TB medication non-adherence. Those patients who had a problem of forgetfulness were seven times more likely to be non-adherent than their counterparts (AOR: 7.04, 95% CI 1.40–35.13). TB patients who were in the continuation phases of chemotherapy were seven times (AOR: 6.95, 95% CI 1.81–26.73) more likely to be non-adherent to anti-TB medications. In addition to this, patients who had symptoms of TB during the interview (AOR: 4.29, 95% CI 1.53–12.03) and had co-infection with HIV (AOR: 4.06, 95% CI 1.70–9.70) were also at higher risk of non-adherence than those who were non-symptomatic during the interview and HIV sero-negatives respectively (Table 4).

**Table 4:** Logistic regression analysis of factors associated with non- adherence to anti-TB (last 1 month).

Characteristics		Non-adherence		Crude OR	Adjusted OR
		Yes	no	(95% CI)	(95% CI)
Forgetfulness	Yes	4	7	5.83(1.59–21.36)	7.04(1.40–35.13)
	No	24	245	1.00	1.00
Phases of Chemotherapy	Intensive phase	3	87	1.00	1.00
	Continuation phase	25	165	4.39(1.29–14.96)	6.95(1.81–26.73)
Symptoms of TB during the interview	Yes	9	27	3.95(1.62–9.59)	4.29(1.53–12.03)
	No	19	225	1.00	1.00
Visited quacks	Yes	7	29	2.56(1.01–6.55)	
	No	21	223	1.00	
Experience side effects	Yes	17	92	2.69(1.21–5.99)	
	No	11	160	1.00	
Co- infection with HIV	Yes	14	50	3.90(1.75–8.71)	4.06(1.70–9.70)
	No	14	195	1.00	1.00
Additional drugs	Yes	9	38	2.67(1.12–6.34)	
	No	19	214	1.00	

North Gondar zone, Northwest Ethiopia, May 2013.

## Discussion

Adherence to anti-TB treatment is a major determinant of treatment outcome. In developing countries where inequities in access to health care are high and health resources are scarce the magnitude and impact of poor adherence is assumed to be higher. It is undeniable that many patients experience difficulties in following treatment recommendations [5], [11]. Hence, this study assessed the level and determinant factors of non-adherence to anti-TB medications.

Though a self-reported level of non-adherence is assumed to be under-estimated, a high proportion (10%) of non-adherence in the last one month before the survey was found in this study. Similarly, a higher proportion of non-adherence (13.6%) in the four days prior to the survey was reported. This finding is more than twice that reported from Kenya (4.8%).

However, the present non-adherence over one month was lower than the previous reports from Southern Ethiopia (20.8%), Uganda (25%), Kolkata, India (40.5%), and the Jiangsu Province of China (12.2%) [12]–[15]. This is probably due to variations in study populations. In this study we included both HIV-positive and HIV-negative patients. The former studies included only TB-HIV co-infected patients. HIV co-infected patients are much more likely to be non-adherent for several reasons. On the other hand, this variation may be attributable to the duration in which the adherence was calculated. In the case of the Ugandan study, adherence was estimated over the last five days and it was over the whole intensive phase in the case of Kolkata India.

In the adjusted analysis; forgetfulness, being in continuation phases of chemotherapy, having symptoms of TB during the interview, and HIV co-infection were significantly associated with anti-TB medication non-adherence. Those TB patients who were in the continuation phase of the chemotherapy were about seven times more likely to be non-adherent than those who were in the intensive/directly observed treatment short-course strategy (DOTS). However, it is believed that DOTS with daily supervision is a difficult task for most patients and in particular HIV co-infected patients who needed to attend the clinics for ART. It is also believed that DOTS is too rigid a strategy [16]. Similar findings were also reported from Southern Ethiopia, Brazil, and Uganda, [13], [17], [18].

TB patients who were symptomatic during the interview were much more likely to be non-adherent than those who did not have symptoms of TB during the interview. Under normal circumstances patients whose TB symptoms were resolved quickly may be urged to leave treatment once they started feeling better. However, in this study the opposite was true. Patients may remain symptomatic as a result of their poor adherence. On the other hand, patients who remain symptomatic while sticking to treatment may be disappointed and intentionally miss their pills. However, a study conducted in Kolkata, India declared that the urge to leave treatment once patient started feeling better was a significant determinant of non-adherence to anti-TB medication [14].

The proportion of HIV co-infection among TB patients was 23.4%. About 69% of these patients were on ART. In this study, HIV co-infected TB patients were less likely to be adherent to their anti-TB medications. HIV/AIDS co-infected patients have many pills to take and the adverse effects of anti-TB medication are more common for HIV co-infected patients [7]. Similarly, HIV/AIDS co-infected patients may be less motivated to take their medication. This phenomenon has been reported in Brazil, Kenya and South Africa [18]–[20].

As previously reported from other studies [12], [21], forgetfulness was also an important problem associated with poor adherence to anti-TB. This problem may be related to older age, as older age was significantly associated with non- adherence over the last four days. This might be also explained by HIV/AIDS related encephalopathy or dementia.

However, previously confirmed factors like distance from a health facility, transportation cost, relationship with health worker, and proper counseling were not associated with non-adherence in this study. Currently, in Ethiopia, TB treatment is being provided freely at the community, grass root level. However, some patients still questioned the accessibility of TB related services. Furthermore, most TB patients had good relationship with their health care provider (99.3%). In general adherence is a dynamic issue and barriers are also liable to change over time, which necessitates continuation of multi-disciplinary collaborative researches. Finally, this study shares the limitations of cross-sectional studies and hence it might suffer from temporal relationship establishment with some variables and might not provide much stronger evidence of causality. In addition to this, this study only addressed a self-reported level of non-adherence and it might be under-estimated.

## Conclusions

Non-adherence among TB patients was high. Forgetfulness, being in continuation phases of chemotherapy, having symptoms of TB during the interview, and HIV co-infection were significantly associated with anti-TB medication non-adherence. Thus, special attention and adherence counseling should be given for those who remained symptomatic, those who are co-infected with HIV, and those in the continuation phase of the chemotherapy. Further research is also recommended since the issue of adherence is dynamic.
